# On detecting and characterizing planetary oceans in the solar system using a distance-based ensemble modelling approach: application to the Uranus system

**DOI:** 10.1098/rsta.2024.0086

**Published:** 2024-12-02

**Authors:** C. J. Cochrane, S. D. Vance, J. B. Biersteker, M. J. Styczinski, B. Weiss

**Affiliations:** ^1^ Jet Propulsion Laboratory, California Institute of Technology, Pasadena, CA, USA; ^2^ Massachusetts Institute of Technology, Cambridge, MA, USA; ^3^ Blue Marble Space Institute of Science, Seattle, WA, USA

**Keywords:** magnetic induction, ocean detection, Umbriel, Uranus moons, Uranus

## Abstract

The discovery of Europa’s subsurface ocean has spawned a strong desire by the planetary community to return and assess the ocean’s habitability using the magnetic induction signal that Europa generates. NASA has since formulated and developed the *Europa Clipper* mission with that same goal, anticipating its arrival in the Jovian system in the early 2030s. In parallel, ESA has developed the *JUpiter Icy moons Explorer* mission to further investigate the interior of Ganymede and other Jovian moons, scheduled to arrive approximately one year later. As a result, extensive work has now been devoted to developing and refining methods to analyse magnetic induction measurements with the goal of characterizing oceans within icy moons, including those in the Neptune and Uranus systems, which are ideal laboratories for such investigations. We present one such method, involving a distance-based inverse and forward modelling approach that leverages self-consistent interior models used to infer ocean and ice-shell properties of various moons that respond inductively to the dynamic magnetic environments in which they reside. We demonstrate the method on a hypothetical ocean within Umbriel, showing the ocean thickness and conductivity constraints that can be inferred from a Monte Carlo error analysis using a three-flyby mission concept.

This article is part of the theme issue ‘Magnetometric remote sensing of Earth and planetary oceans’.

## 1. Oceans in the solar system

Water is abundant in the solar system, with several moons each harbouring more extant liquid water than in all of Earth’s oceans, lending promise to the search for habitable worlds beyond our own planet [1]. Detecting and characterizing these potential oceans in planetary bodies is of great interest to the scientific community and to the public. One promising and proven technique to detect subsurface water and hidden oceans is to use electromagnetic sounding, which relies on either active or passive probing methods. Active methods involve transmitting pulsed or chirped electromagnetic waveforms as the source (e.g. ice penetrating radar, see [2,3] for sounding of ice masses on Earth and Mars, respectively), while passive methods leverage naturally occurring sources to sound for their reflected or excited signals, some including the very slow (≪ 1 Hz) magnetic field variations exhibited by planetary magnetospheres arising from relative planetary motion [4,5] and the higher-frequency electromagnetic radiation radio emissions resulting from magnetic-field-accelerated electrons from the Sun [6] or even large planets like Jupiter [7,8] and Uranus [9]. Geodetic methods might be used to search for oceans within moons with strong gravitational forcing, enabling the measurement of Love numbers by tracking the acceleration of a spacecraft flying by the moon [10]. Forced physical libration in longitude is another way to detect the presence of a deep ocean [11].

For the Uranus system, where our method is applied, passive sounding using natural magnetic sources serves as the most robust method to search for oceans within the moons owing to the large dynamic field that is present. The passive magnetic sounding method has already proven promising in the Jovian system, where magnetic field measurements from the *Galileo* mission provide the strongest evidence for subsurface liquid-water oceans under the ice shells of Europa and also Callisto [4,12,13]. These global bodies of water contain dissolved salts in chemical equilibrium with their rocky interiors, making the oceans electrically conductive. This feature makes passive magnetic induction investigations of their properties possible because the oceans reside in time-varying magnetic fields, arising from the tilt of Jupiter’s magnetic field and the slight orbital eccentricities and inclinations of the moons. Although Ganymede also creates its own intrinsic magnetic field, it is also expected to host a subsurface ocean [14], with strong evidence from analysis of the UV aurora at Ganymede [15]. Further measurements by ESA’s *JUpiter ICy moons Explorer (JUICE*) will be able to confirm or refute the presence of this ocean because of the greater spatial and temporal coverage needed to disentangle any potential buried induction response from its static internal dynamo-generated magnetic field [16].

It is likely that Saturn’s moon Enceladus also has a global subsurface ocean, as evidenced by the plumes from its south pole and its libration state [17]. However, the ocean has not been confirmed by magnetic induction measurements acquired by NASA’s *Cassini* spacecraft because the small expected induction signal of approximately 4.8 nT owing to orbital eccentricity [18] is overwhelmed by the field signature associated with the diversion of ionized particles around the neutral plume ejecta [19]. Thus, any future magnetic investigation of an ocean at Enceladus would need to accurately characterize plasma processes to disentangle the weak induction response or be measured on the surface free of the obscuring plume signature.

Mimas is the only other moon of Saturn with an excitation field strong enough to be viable for passive magnetic sounding. Its eccentricity is expected to drive variations in the external magnetic field by less than 43.2nT in its true anomaly period (close to its orbital period) [18]. Although Titan also exhibits an eccentric orbit, it is too far from Saturn to produce a detectable signal from its possible ocean.

The moons of the ice giant planets Uranus and Neptune also provide great targets for magnetic induction investigations because of the strong and dynamic magnetic fields in which they are immersed. The magnetic fields of both planets are especially unique as they have extremely tilted magnetic axes with respect to their spin axes ($59^{\circ }$ for Uranus and $47^{\circ }$ for Neptune) and they also have relatively large quadrupole moments (and therefore stronger field gradients) compared with their dipole moments. Although *Voyager 2* did not pass close enough to any of the moons on its flybys of these systems for magnetic sounding investigations, recent analysis demonstrates that magnetic induction would be an ideal tool for exploratory missions to detect and characterize possible oceans in the five large moons of Uranus [20–22] and in Neptune’s moon Triton [23,24].

Here, we revisit the recent magnetic induction analyses at Uranus to demonstrate the ability to magnetically detect and characterize an ocean in the moon Umbriel through a novel distance-based ensemble modelling approach for a previously developed Discovery mission concept called *Urania*.

## 2. Inferring ocean properties from magnetometric observations

**Figure 1.**
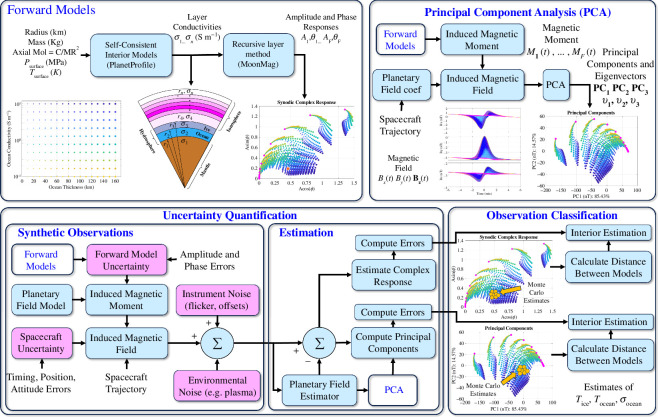
Block diagram of our distance-based model ocean detection and characterization approach, including a forward modelling chain, uncertainty quantification using Monte Carlo error analysis for synthetically generated observations and observation characterization. Using this data processing chain, two different methods can be employed to infer interior properties from measurements using self-consistent models produced by the forward modelling: (1) inversion for the amplitude and phase delay of the complex response function at multiple frequencies or (2) projection of the eigenvectors obtained from PCA on to an alternative basis for classification.

The relationship between the induced magnetic field from the internal conducting layers and the characteristics of those layers is non-unique and nonlinear. To map magnetic induction measurements to plausible moon interior profiles, one must compare the induced field predicted from a hypothesized interior conductivity structure (e.g. forward modelling) with that inferred from the observations (e.g. least-squares inversion, principal component analysis (PCA), Bayesian inference, etc.). Figure 1 illustrates an entire processing chain that can be used to achieve this goal, the three main blocks of which include forward modelling, uncertainty quantification and observation classification.

### (a) Forward modelling

For a planetary profile with radially varying and spherically symmetric conductivity, the magnetic diffusion equation may be solved analytically by a layer method [25–27], and the result is a magnetic dipole moment proportional to the excitation field at each oscillation frequency. The constants of proportionality constitute the complex response function $A^{e}$, and are typically represented by a set of frequency-dependent induction amplitudes A and phase delays ϕ. For a perfectly conducting sphere the size of the body, A=1 and ϕ=0. Past studies have typically focused on either fitting dipole moments to magnetometer data to infer $A^{e}$ [28,29], or forward models comparing the induction amplitude from considered interior structure hypotheses to the values inferred from data [26,30].

Because the induced magnetic field relates to the radial profile of electrical conductivity and no other physical properties of the body, some assumptions about the hypothesized interior possibilities are required. Such assumptions are often informed by converging lines of evidence arising from non-magnetic investigations, as in the case of gravitational field measurements used to infer the bulk density and axial moment of inertia (MoI). The synthesis of complementary information increases the robustness of constraints on the interior properties inferred from the measurements when self-consistency is enforced. Self-consistency is a harmonious relationship between all model inputs, assumptions and outputs, such that there is minimal internal inconsistency. For example, the MoI inferred from gravity data for Europa [31,32] suggests a hydrosphere thickness of 80−170km. The salinity w and the composition of dissolved salts in any modelled ocean will affect the MoI value, such that a combination of a thin ice shell and a thick ocean with a high salt content may require a rocky mantle with certain properties to match the total bulk density that prevents matching of the MoI under these conditions. Such a model would not be self-consistent. Although self-consistent models are more difficult to create, inversion of magnetic measurements using them offers the most robust ability to combine as much information as possible from complementary investigations in sounding studies.

Our work uses the PlanetProfile framework [33,34] to build self-consistent spherically symmetric forward models for ocean worlds. PlanetProfile uses established and custom software packages to evaluate the equations of state for hypothesized materials in ocean planetary bodies, including ice [35], rocks [36,37] and ocean composition [38,39] at each temperature T and pressure P for each material layer, propagated downward from the surface in discrete steps, tracking ambient conditions such as local gravity. Input assumptions such as the rock composition, ocean salt composition and melting temperature, the tidal heating rate, and character of the thermal profile (e.g. parameters for solid state and fluid convection) are applied to make the problem tractable and to generate the parameters used in a distance-based model approach. For the present work, we use the same models generated for our other recent study of the Uranus system [21], using the MATLAB implementation of PlanetProfile to compute the magnetic induction responses for simple three-layer Umbriel interiors. We vary the thickness and electrical conductivity of the ocean atop a non-conducting solid interior, with varying thicknesses of overlying ice. We also account for the influence of an overlying ionospheric layer.

**Figure 2.**
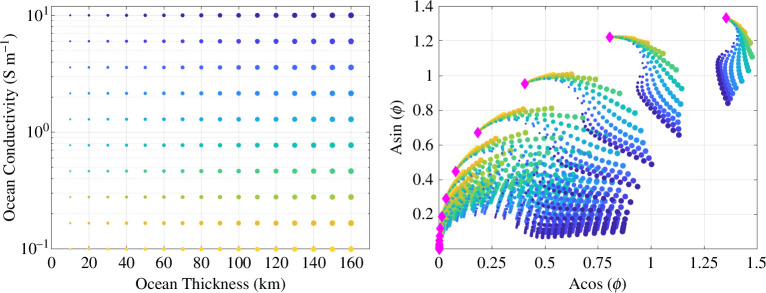
The set of forward models for Umbriel used in this study. (left) Ocean model parameters considered in this study, with specific combinations of thickness and conductivity. The hydrosphere thickness (i.e. seafloor depth) of 165km is assumed for all models. This set of 160 ocean models is convolved with every 21 ionosphere conductance cases, resulting in 3381 total models (which includes the 21 ionosphere-only models also considered). Note that the size of the dot corresponds to ocean thickness and the colour is based on ocean conductivity. (right) Complex response coefficients at the synodic period for each of the 3381 models , where the colour and size of the ocean models are consistent with the figure on the left. Magenta diamonds in this case represent the induction response at the synodic period from ionosphere-only models (i.e. no ocean) for various ionosphere conductances.

The left-hand panel of figure 2 illustrates the representative forward modelling space used in this work for Umbriel, showing the various combinations of ocean thickness (from 0 to 160km in 10km steps) and ocean conductivity (from 0.1 to 10S/m in log steps), for an assumed hydrosphere thickness (i.e. seafloor depth) of 165km. The right-hand panel of figure 2 illustrates the complex response function of those models evaluated at the apparent 20.8h synodic rotation rate of Uranus. Similar plots can be made for the orbital period, in addition to the beats and harmonics of these two fundamental periods, but excitations at these frequencies are relatively weak for Umbriel so they are not shown here. We also include the effects of a small conductive ionospheric layer for various values of height-integrated conductivity or conductance, from 0.1S to 5000S in linear-log steps, for each ocean model considered. Ionosphere-only models are represented by magenta diamonds, and the cluster of ocean-bearing models they are associated with are colour-coded and sized based on the ocean-model parameter space shown in the left-hand panel. Because Umbriel is a relatively small moon with small gravitational effects, it is probably unable to sustain a significant ionosphere, so the effects will be small. In total, we assess 3381 models in this study. The large orange dot in the right-hand panel represents the single-ocean case from which we generate our synthetic data to test our two retrieval methods. The axes in this case $A_{R,k}=A_{k}\cos(\theta_{k})$ and $A_{I,k}=A_{k}\sin(\theta_{k})$, where the subscripts R and I represent the real (in phase) and imaginary (quadrature or out of phase) components. We illustrate the models in this space because it is precisely these coefficients which can be linearly separated from the time-dependent terms in the induction response and therefore can be directly obtained from the inversion methods described in §2c(ii).

### (b) Synthetic observations

We demonstrate the importance of uncertainty quantification for ocean detection and how it relates to the constraints that can be made on the estimations of the interior properties of the moon. Using a Monte Carlo approach which leverages a large synthetic observation dataset with injected errors from various sources, as shown in figure 1, we can estimate the uncertainty in the ocean parameters resulting from errors with different methods. The methods investigated in this study include a data inverse approach and a PCA approach, both showing comparable performance for the application at hand.

We create a synthetic dataset with relevant simulated errors to determine how well intrinsic parameters can be extracted from noisy observations. We use the amplitude and phase responses from the plausible ocean models of Umbriel shown in figure 2 to help forward model the induced magnetic moment using the analytical expression described by [28],


(2.1) $$\begin{array}{ccc} & 𝐌=ℜ\left\{-\frac{4\pi }{\mu_{o}}\frac{R^{3}}{2}Ae^{i\phi }{𝐁}_{U}(t)\right\}=-\frac{4\pi }{\mu_{o}}\frac{R^{3}}{2}𝐦(t), & \end{array}$$

in units of $A\,m^{2}$, where 𝐦(t) represents the time-varying portion of the induced magnetic moment, a scaled and time-delayed version of the driving field of Uranus in the synchronous frame of the moon, with units of nT with the resulting time-dependent induced magnetic field,


(2.2) $$\begin{array}{ccc} & {𝐁}_{\mathrm{Ind}}=\frac{\mu_{o}}{4\pi }\frac{3(𝐫\cdot 𝐌)𝐫-|𝐫{|}^{2}𝐌}{|𝐫{|}^{5}}. & \end{array}$$

Here, R is the radius of the moon, 𝐫=[x,y,z] is the position vector, ${𝐁}_{U}(t)$ is the planetary driving field and $Ae^{i\phi }$ defines the complex response function of the ocean, with amplitude A and phase delay ϕ.

**Figure 3.**
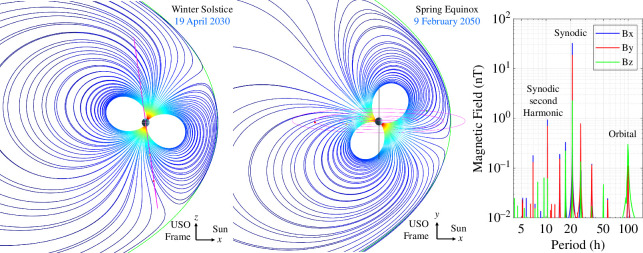
Magnetic field of Uranus. (left) Magnetic field line geometry of Uranus for Winter Solstice and Spring Equinox in the Uranus–Sun–Orbit frame. The orbits of Miranda, Ariel, Umbriel, Titania and Oberon are shown. Independent of season, the magnetic field of Umbriel (as well as Miranda and Ariel) is well shielded from external effects of the magnetosphere, making it a robust target for magnetic induction investigation of its interior (right) Fourier transform of the magnetic field of Uranus evaluated at the position of Umbriel. Note that although there are many beats and harmonics of the two fundamental frequencies, only the synodic is used here for characterization owing to its dominance over the other frequencies.

As detailed in many previous works [20–22], the source of the driving field for magnetic induction at the uranian moons arises from the complex magnetic geometry, where variations in the magnetic field are predominantly attributed to the tilt in its magnetic axis with respect to its spin axis and therefore encounter variations in magnetic latitude as Uranus rotates. However, some moons experience small perturbations at their orbital periods owing to their slightly eccentric and inclined orbits or even seasonal perturbations attributed to solar wind magnetosphere interactions such as magnetopause currents [40], magnetic reconnection [41] or Kelvin–Helmholtz instabilities [42]. The seasonal shape of the magnetosphere, modelled after [20], is illustrated in figure 3, showing both the winter solstice and spring equinox configurations. Note that depending on season, the outer uranian moons Titania and Oberon could be influenced by these perturbations, although recent reanalysis of *Voyager 2* data suggests that the magnetopause may be farther away than originally anticipated, suggesting that ocean detection in these outer moons may be more feasible [43]. In addition, the inner three large moons, Miranda, Ariel and Umbriel, are insulated from these external effects, making them promising targets for magnetic induction investigation of their interiors.

The right-hand panel of figure 3 illustrates the spectral attributes of Uranus’ magnetic field in the frame of Umbriel as computed from the AH_5_ magnetic field model [44]. We chose to assess ocean detection on this moon because recent evolutionary modelling indicates that it may harbour a subsurface ocean [45]. The intrinsic field model defines the planetary driving field in the frame of the moon in our synthetic dataset ${𝐁}_{U}(t)$. The intrinsic field model is also used to define the magnetic field in the reference frame and the position of the spacecraft ${𝐁}_{U,SC}(t)$. To inject errors into the measurements on each flyby, we first randomly scale the complex response coefficients by a small configurable amount to account for uncertainties in the forward models such as small asymmetries in ocean layer shape. We also add a spacecraft position uncertainty by randomly shifting the position vector 𝐫(t)=[x(t),y(t),z(t)] and a timing uncertainty by adding a small random Δt to the ephemeris time, each by a constant amount on each flyby. We also inject attitude uncertainty by slightly rotating the resulting magnetic field vector and also add magnetic field offset uncertainty ${𝐁}_{o}(t)$, attributed to sensor offsets and spacecraft magnetic field, and instrument noise ${𝐁}_{n}$ to each axis. The total synthetic observation field is defined by


(2.3) $$\begin{array}{ccc} & {𝐁}_{SC}(t)={𝐁}_{U,SC}(t)+{𝐁}_{\mathrm{Ind}}(t)+{𝐁}_{n}(t)+{𝐁}_{o}. & \end{array}$$

The main error source that we neglect to include in this study is that from the field effects that arise from magnetospheric or ionospheric plasma currents. These effects can only really truly be simulated with magnetohydrodynamic (MHD) or hybrid kinetic modelling which are very computationally resource and time expensive, making it very difficult to integrate with the Monte Carlo error analysis that we set out to perform in this work. Even the use of a single simulation is not very informative as it biases or offsets the interpretation of interior properties [23]. However, recent advances in machine learning may soon be able to emulate these types of signals so that this systematic noise component can be integrated into our Monte Carlo error analysis. For example, in Ref. [46] a Gaussian process emulator was produced which successfully replicates the MHD simulation of the Juno PJ34 flyby of Ganymede [47]. Future work is expected to integrate such methods to better capture the influence of plasma signatures on the uncertainty of ocean characterization.

### (c) Estimation methods

To best infer the interior properties from the synthetic observations, or real measurements once they are collected, one must first disentangle the field contributions. The spacecraft field is usually removed first using a gradiometry approach [48–50], where at least two sensors are available to measure the spacecraft field gradient, which separates it from the background field (assumed to be uniform across the sensors) to be measured. Next, environmental effects such as plasma interaction fields must also be removed, but these are not considered in the scope of this work for reasons already described above. The planetary background magnetic field is then separated from the induction signal to isolate each field contribution for further processing. Finally, estimation methods are used to infer interior properties from the isolated induction signals.

#### (i) Planetary field estimation via data inversion

**Figure 4.**
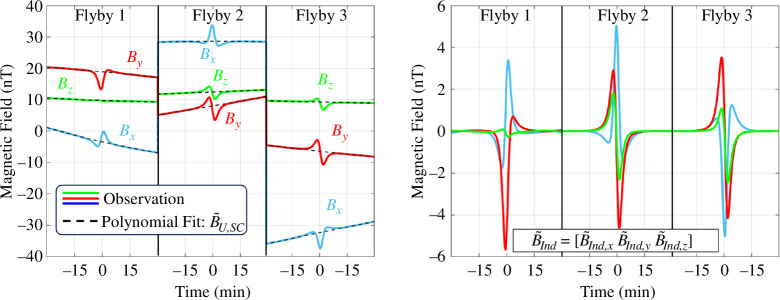
Prepossessing of the observations from a multi-flyby mission concept. (left) Observations plotted against the second-order polynomial fitting routine used to separate the background field from the induction signal. (right) The resultant induction component after the background field is subtracted off.


To isolate the planetary magnetic field from the measurements, we use a least-squares-fitted second-order polynomial to approximate the slowly spatially varying field at the position of the spacecraft ${\tilde{B}}_{U,SC}(t)$ from the faster spatially varying induction signal ${\tilde{B}}_{Ind}(t)$ for a small window of time centred about the closest approach to the moon on each flyby. Figure 4 conceptually illustrates this step for data acquired from the *Urania* multi-flyby mission concept (see §3). The approximated background fields at the position of the spacecraft on each flyby are jointly used to estimate the magnetic field of the planet at the position and frame of the moon with

(2.4) $${\tilde{B}}_{U}(t)={\tilde{B}}_{U,SC}(t)-B_{U,SC,m}(t)+B_{U,m}(t),$$

where the last two contributions, ${𝐁}_{U,SC,m}(t)$ and ${𝐁}_{U,m}(t)$, are the magnetic field estimates from the AH_5_ model [44] used to correct for the slight differences in magnetic fields anticipated to be present at the spacecraft and moon (see e.g. [13,51]). As illustrated in the right-hand panel of figure 3, the magnetic field in the synchronous frame of the moon is composed of a series of tones and therefore can be modelled as a superposition of sinusoids:


(2.5) $$\begin{array}{ccc} & {\hat{𝐁}}_{U}(t)=\sum_{k}{𝐁}_{R,k}\cos\left(2\pi f_{k}t\right)+\sum_{k}{𝐁}_{I,k}\sin\left(2\pi f_{k}t\right), & \end{array}$$

where ${𝐁}_{R,k}=[B_{R,k,x},B_{R,k,y},B_{R,k,z}]$ and ${𝐁}_{I,k}=[B_{I,k,x},B_{I,k,y},B_{I,k,z}]$ are the model parameters and each contain three parameters (e.g. for the x, y and z axes) for each frequency k of interest, including a DC term where $f_{k}=0\;Hz$. Again, the subscript R indicates the real (in phase) component and I the imaginary (quadrature or out of phase) component. The amplitudes are therefore ${𝐁}_{k}=\sqrt{|{𝐁}_{R,k}{|}^{2}+|{𝐁}_{I,k}{|}^{2}}$ and phases ${𝛉}_{k}=\tan^{-1}({𝐁}_{I,k}⊘{𝐁}_{R,k})$, where ⊘ is the element-wise division or Hadamard operator, are reference to the J2000 epoch. We minimize the sum of squared errors between ${\tilde{B}}_{U}(t)$ and ${\hat{𝐁}}_{U}(t)$ using a linear least-squares inversion approach to acquire a set of coefficients $B_{R,k}$ and $B_{I,k}$ for all relevant frequencies k for each of the three axes—the excitation moments. The modelling approach is described in detail by [18]. Owing to the uncertainty in the temporal variation of Uranus’ multi-polar field, it is important that future missions design trajectories such that flybys of the same moon are spaced as close in time as possible to reduce potential drift of the driving field.

#### (ii) Interior property estimation via data inversion

One method that enables ocean property estimation entails solving for the complex response parameters at multiple frequencies using an inverse data approach. Solving for the amplitudes and phase delays of the induced field directly allows one to link the observation to interior properties based on similarity to the forward-modelled dataset. We have demonstrated this method for the inversion of the complex response function over multiple flybys of Callisto using *Galileo* data to provide more compelling evidence that an ocean is more likely to be responsible for the induction response than an ionosphere alone [13].

Because the driving field can be represented by a superposition of sinusoids of different frequencies (equation (2.5)), an induced magnetic moment of degree 1 (a dipole) will be generated within the ocean that is synchronized with all driving frequencies, but scaled in amplitude and delayed in time. The time-varying portion of the dipole magnetic moment 𝐦(t) of equation (2.1) can therefore be approximated by the following model:


(2.6) $$\begin{array}{ccc} & \hat{𝐦}(t)=\sum_{k}A_{k}{𝐁}_{k}\cos\left(2\pi f_{k}t+{𝛉}_{k}-\phi_{k}\right), & \end{array}$$

and the associated induced magnetic field can be modelled by


(2.7) $$\begin{array}{ccc} & {\hat{𝐁}}_{\mathrm{Ind}}(t)=𝐃(t)\hat{𝐦}(t)=𝐃(t)𝐏(t)𝐀, & \end{array}$$

where the complex response model parameter array $𝐀=[A_{R,1},A_{I,1},\ldots ,A_{R,K},A_{I,K}]$ for K frequencies are linearly separable from the time-dependent terms 𝐃(t) and 𝐏(t). This formulation is essentially identical to equation (2.2), but is in matrix form with isolated induction coefficients. Here, 𝐃(t) is the time-dependent dipolar matrix which mixes the magnetic moment components into each axis of the magnetic field and 𝐏(t) is the time-dependent matrix that contains the planetary driving field coefficients ${𝐁}_{R,k}$ and ${𝐁}_{I,k}$ for each flyby, defined and derived, respectively, in the Appendix. We leverage a linear least-squares approach which minimizes the sum of squared errors between ${\hat{𝐁}}_{\mathrm{Ind}}(t)$ and ${\tilde{B}}_{Ind}(t)$ to acquire a set of induction parameters 𝐀 that is optimal across multiple flybys. The full set of equations are derived in the Appendix for completeness.

#### (iii) Interior property estimation via PCA

A second way to infer ocean properties from magnetometric measurements is through a method known as PCA. This method is a dimensionality reduction routine that is able to uncover hidden patterns and trends of a large dataset with high dimension, such as the induced magnetic field time series associated with our numerous forward models. In this application, forward models are used to create a set of induced magnetic moments and induced magnetic field time series along the trajectory using the planetary driving field coefficients (see figure 1). The samples from the time series of each flyby are first concatenated by component (e.g. $B_{x}$, $B_{y}$ and $B_{z}$), then the resultant three arrays are further concatenated to form a large contiguous set of samples that represent the induction signatures for each model throughout the mission. Assuming for simplicity that there are N samples for each component in each of the F flybys, each resultant time series consists of an array of 3NF samples in length. These arrays are stored in the columns of a large matrix, which is then centred (i.e. the mean across each row is removed), to prevent the method from retrieving principal components (PCs) that reflect the mean of the dataset rather than those associated with the direction of maximum variance. Then, eigenvalue decomposition or singular value decomposition is performed to extract a set of eigenvalues 𝛌 and eigenvectors 𝐕 that can project the data on to a space of reduced dimensionality (the PCs). If the background-field-removed observations ${\tilde{B}}_{Ind}$ are concatenated in a similar fashion to that of the forward modelled matrix, as in


(2.8) $$\begin{array}{l} B_{Ind,F}=[{\tilde{B}}_{Ind,x}(t_{1})\ldots {\tilde{B}}_{Ind,x}(t_{NF}),{\tilde{B}}_{Ind,y}(t_{1})\ldots {\tilde{B}}_{Ind,y}(t_{NF}),{\tilde{B}}_{Ind,z}(t_{1})\ldots {\tilde{B}}_{Ind,z}(t_{NF}){]}^{T}, \end{array}$$

then the eigenvectors 𝐕, each of length 3NF, can be used to project the noisy observations into the PC space via the simple operation,


(2.9) $$PC=V^{T}{\tilde{B}}_{Ind,F}.$$

In most applications, the first three eigenvectors and PCs, e.g. $𝐏𝐂=\left[PC_{1},PC_{2},PC_{3}\right]$, are sufficient for characterization, especially in the system under consideration where a single magnetic wave drives induction. The PCs associated with the observations can then be compared with the PCs of the forward models to assess ocean properties, as described in the next section.

At the cost of slightly increased processing time, the PCA approach allows for much simpler implementation and interpretation of the observations. The dimensionality reduction and parameter space conversion associated with PCA is sometimes viewed as a black-box that is widely used but is often misunderstood. However, in our application, we have previously derived the direct relationship between the PCs and the induced magnetic moments that define them. See [23] for this derivation and a more in-depth description of this method for ocean detection in Neptune’s moon Triton.

### (d) Observation classification

To gain insight into the nature of the inverted complex response coefficients or PCs, they are compared with the forward model parameter suite via distance-based calculations. For the inversion approach, we calculate the Euclidean distances between the estimated coefficients $A_{R,k}$ and $A_{I,k}$ acquired from the observations and all forward model coefficients $A_{R,k,j}$ and $A_{I,k,j}$ with model index j and frequency k such


(2.10) $$D_{j}=\sqrt{\sum_{k}(A_{R,k,j}-A_{R,k}{)}^{2}+(A_{I,k,j}-A_{I,k}{)}^{2}}.$$

Note that the distance metric is unitless as it is constructed in the complex plane. The separation distances associated with the forward models can then be plotted as interpolated contours in the ocean thickness versus ocean conductivity plane to determine the set that is most similar to those estimated from observation. The interior properties (e.g. ice-shell thickness, ocean thickness and electrical conductivity) associated with these closely spaced models therefore provide the range of possible interior configurations that are capable of producing the observations. For the case of Umbriel, we only use the complex coefficients associated with the synodic period owing to their dominance over the other coefficients, and therefore k identifies the frequency associated with the 20.8h period. However, in systems that have strong orbital components, a hyperdimensional space can be formed to improve separation of like-model classes and hence characterization ability (e.g. Europa and Triton). In this case, it is recommended that the separation distance be calculated based on the induced magnetic moments (see equation (2.6)) as performed by [21], or by weighting the induction coefficients in equation (2.10) by the strength of the periodic fields that drive them,


(2.11) $$\begin{array}{ccc} & D_{j}=\sqrt{\sum_{k}B_{k,max}^{2}\left[(A_{R,k,j}-A_{R,k}{)}^{2}+(A_{I,k,j}-A_{I,k}{)}^{2}\right]}, & \end{array}$$

which has units of nT. Here, $B_{k,max}$ is the maximum field component in the planetary field vector ${𝐁}_{k}$. Analogously, the separation distance between the estimated PCs 𝐏𝐂 and the PCs from the set of forward models ${𝐏𝐂}_{j}$ are computed by


(2.12) $$\begin{array}{ccc} & D_{j}=\sqrt{\sum_{i}(PC_{i,j}-PC_{i}{)}^{2}}, & \end{array}$$

where the index i represents the PC, here i=1,2,3 and j represents the model index in the forward model dataset. The unit of separation in the PC space is also in units of nT.

For one to determine which ocean models in the modelling space are discernible from the single-ocean model under study, a Monte Carlo analysis is performed to assess the uncertainty in parameter space from increased noise and spacecraft uncertainties. The statistics associated with the acquired errors are essential for identifying the constraints that can be placed on the ocean parameter space. Models that lie within a 3σ error are considered indistinguishable from the model under study, and therefore interior properties cannot be distinguished within these bounds. The constraints can generally be tightened by reducing instrument noise and spacecraft uncertainties, increasing the number of flybys and increasing the number of additional frequencies used in the analysis. It is also important to note that this separation metric is used to estimate the amount of margin that is needed to distinguish an ocean response from that of a purely ionospheric response [13,21,23]. However, this metric is less of a concern in the case of Umbriel than in the cited studies because Umbriel is a relatively small moon and, therefore, less likely to harbour a strong ionosphere capable of producing an induced magnetic field.

## 3. Case study: *Urania* mission concept

**Figure 5.**
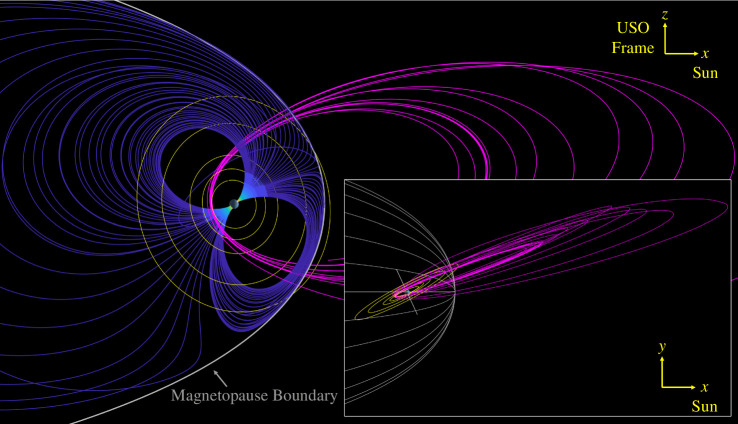
Trajectory superimposed on magnetic field lines of Uranus for the epoch 9 March 2044 20:00:00 UTC. This trajectory contained two flybys of Miranda, two flybys of Ariel, three flybys of Umbriel, two flybys of Titania and three flybys of Oberon and multiple magnetopause crossings to maintain these flybys.

We demonstrate the described methods using the trajectories developed under the *Urania* Discovery concept. Named after Uranus’ granddaughter and the muse of astronomy, *Urania* was a multiple flyby Uranus orbiter concept developed at the Jet Propulsion Laboratory in 2021−2022. The concept had primary aspirations to detect oceans within the moons of Uranus, since they all show clues, in one form or another, of the possibility of harbouring liquid water beneath their icy shells [52–56]. Various trajectories were developed to enable multiple flybys of the major moons. The one used in this work entails three Miranda flybys, two Ariel flybys, three Umbriel flybys, three Titania flybys and two Oberon flybys, making orbits of Uranus approximately in-plane with the orbital plane of the moons as illustrated in figure 5. At least two flybys were chosen for each moon to distinguish the induced magnetic fields from intrinsic magnetic fields the bodies may possess. In the figure, the trajectory is superimposed on the magnetic field lines of Uranus, which are evaluated on 9 March 2044 20:00:00 UTC near when the spacecraft would arrive at the Uranus system. This also corresponds to a time when the xz-planes of the Uranus–Solar–Magnetospheric (USM) frame and Uranus–Solar–Orbital (USO) frame coincide.

To demonstrate the effectiveness of the described methodologies, we use this trajectory to determine how well we can resolve a single ocean model within Umbriel after three flybys among the thousands of plausible interior scenarios. Note that the altitude at the closet approach of each flyby is 100km and the associated Uranus east longitudes ($193.9^{\circ },127.2^{\circ },299.4^{\circ }$) provide adequate sampling of the synodic phase space to model the dominant synodic wave. The synodic complex response coefficients of this ocean are plotted as an orange dot among the 3381 models, as illustrated in figure 2.

We perform Monte Carlo simulations, each three-flyby mission concept considered as a single epoch, with a magnetometer sample rate of $F_{s}$ = $0.2\;s^{-1}$ (i.e. sampling period of 5s), performed 1000 times so that error statistics could be gathered for both methodologies. For each flyby of each epoch, we randomize the noise and uncertainties using an error model that includes ±0.1nT random instrument noise, ±5nT offset uncertainty (which includes residual sensor offsets and spacecraft field), $\pm 5^{\circ }$ attitude uncertainty, ±10km position uncertainty, ±1s timing uncertainty and ±10% forward modelling parameter error, all specified to a 3σ normal distribution. These errors are relatively large for those expected from a standard scientific mission, but used here to simply better demonstrate the noise performance of the method and to capture worst-case scenarios. Spacecraft uncertainties are usually better by an order of magnitude or more in all categories: attitude uncertainty $\leq {\pm 0.1}^{\circ }$, position of ≤±1km and timing uncertainty ≤±1ms.

**Figure 6.**
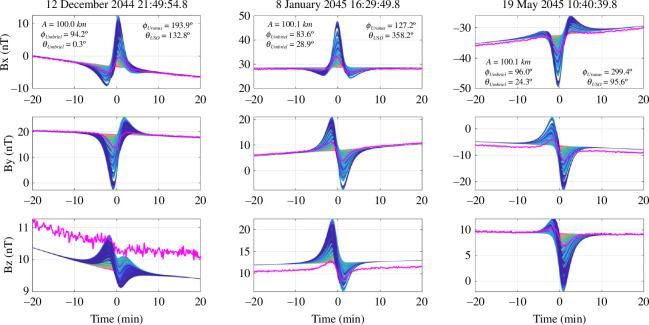
Forward modelled magnetic field along the *Urania* trajectory for the three flybys of Umbriel. A representative set of the noisy synthetic magnetic field observations, coloured magenta, generated for each of the three flybys, plotted on top of the true magnetic responses from all forward models considered in this study (shades of blue). Note that the apparent offset in the synthetic observations with respect to the forward modelled data is attributed to the injected angular uncertainty and offsets.

For the field line correction, the AH_5_ magnetic field model [44] is used as the true planetary field in the synthetic observations, while we assume the dipole-only portion of this model for the field line correction applied in equation (2.4) to account for uncertainty in the planetary field. Because *Voyager 2* only made a single pass of Uranus, there is ambiguity in the strength of its quadrupole and octopole moments which are partly responsible for the synodic field harmonics experienced by the moons. Therefore, our inversion model includes the DC background field and the synodic period, and also the synodic second harmonic to account for the possible presence of this period. We do not invert for the magnetic field with associated orbital period because it is expected to be weak owing to the near-circular orbit of Umbriel. Figure 6 illustrates a representative noisy synthetic dataset based on our representative ocean, plotted against the induced field signature associated with all 3381 forward models, colour-coded based on the scheme illustrated in figure 2.

**Figure 7.**
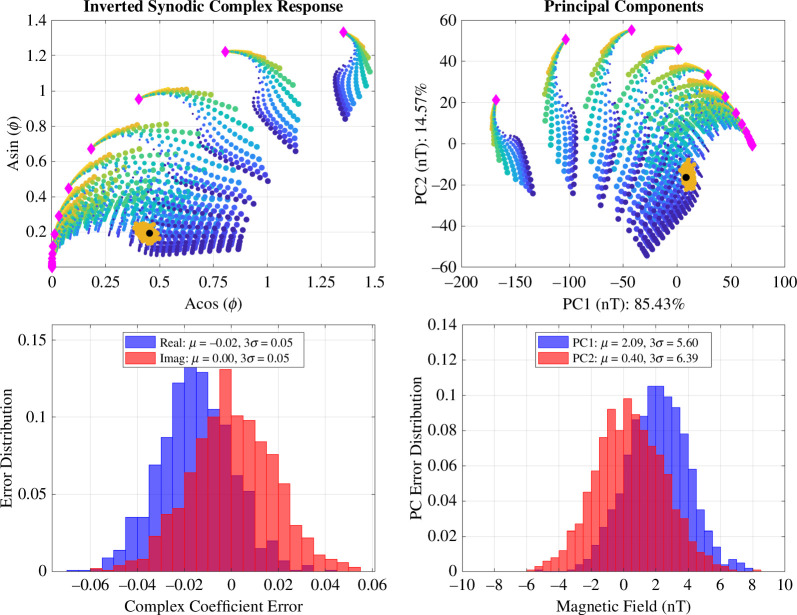
Results of the inversion and PCA Monte Carlo analysis. (upper left) Inverted synodic complex responses from the Monte Carlo simulation (orange), plotted on top of the synodic complex response from all forward models used in this study. The black dot represents the true model response used in this study for reference. The colours associated with the large set of ocean-plus-ionosphere and ionosphere-only models are consistent with that in figure 2. (bottom left) Errors associated with each iteration of the Monte Carlo simulation from the inversion approach. (upper right) PCs associated with the Monte Carlo simulation (orange), plotted on top of the PCs from all forward models used in this study. The black dot again represents the true model response used in this study for reference. (bottom left) Errors associated with each iteration of the Monte Carlo simulation from the PCA approach.

Figure 7 illustrates the classification spaces for (left) the synodic complex plane and (right) the PC space. The location of the ocean under study is marked by the black dot and the orange ‘fuzz’ surrounding the dot represents the estimates from the 1000 Monte Carlo epochs, for both the inverted parameters in the synodic plane and projected measurements into the PC space, respectively. The bottom two panels of figure 7 illustrate the errors of each method. As illustrated, the 3σ spread of errors for inversion are 0.05 (unitless) and 6nT for the PC space. The spread of these errors defines the distinguishability between oceans that have similar responses.

## 4. Discussion

**Figure 8.**
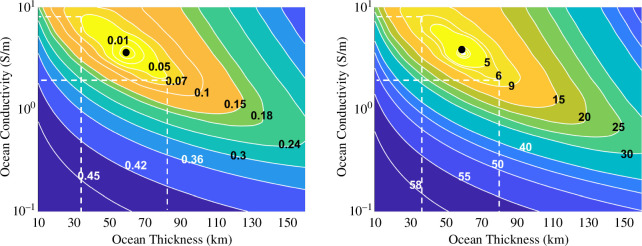
Contours representing the ocean separation distance for retrieval of the test case ocean model used in this study (ocean thickness 60km, ocean conductivity 3.5S/m, ice-shell thickness 105km, ocean depth 165km, ionospheric conductance 1385S) from all other oceans used in this study in the (left) synodic complex response plane and the (right) PC space. Note that because both methods used identical synthetic data, they result in similar classification performance of underlying ocean parameters. As illustrated in figure 7, the 3σ spread of noise is 0.05 in the synodic complex space for inversion and 6nT in the PCA space, respectively. These error regions correspond to identifying the ocean thickness to within 35−85km and conductivity within 2−9S/m.

Recovery of amplitude and phase parameters of the induction field can be robustly extracted from the magnetometer measurements using the two methods described. However, to relate these extracted parameters to the properties of the ocean, one must calculate the separation from the model under study against all forward-modelled datasets. Figure 8 illustrates the separation contours in the synodic complex plane and PC space, respectively, calculated from interpolated distances from our representative ocean case with all other discrete ocean cases in the forward modelled space, for an ionospheric conductance of 100S. For this study, we fixed the ionosphere conductance to demonstrate the effects on uncertainty caused by this parameter. In the synodic complex plane, the ocean models that lie within the 0.05 (3σ) contour of the representative ocean are indistinguishable from each other. This is because the 3σ spread in the errors obtained from the Monte Carlo sampling quantifies the parameter’s uncertainty, which ties directly to interior property constraints based on the forward-modelled space. Similarly, for the PC space, the ocean models that lie within the 6nT (3σ) contour of the representative ocean are indistinguishable from each other based on the errors obtained from the PCA Monte Carlo simulations. Note that both methods produce very similar characterization abilities. In both classification spaces, the representative 60km thick ocean can be characterized to within a thickness of ±25 km (35−85km) and conductivity within 2−9S/m after three flybys. We achieve similar results using a Bayesian inference approach [57], a method previously applied to Europa [51]. Note that the level of noise and spacecraft uncertainties assumed in this analysis is pessimistic, specifically the offset uncertainty (±5nT, 3σ) and the angular uncertainty ($\pm 5^{\circ }$, 3σ), mainly to more dramatically show the effect of these errors. However, the offset uncertainties of ±1nT and the angular uncertainty of $\pm 1^{\circ }$ are probably attainable and would place tighter constraints on the ocean properties. Another way to tighten constraints is to add more flybys to the trajectory, as doing so has the tendency to reduce the region of degeneracy in the classification space.

We note the importance of having at least three flybys for any inference on characterization if the driving field is to be solved, especially for the Uranus and Neptune environments where existing models are derived from very few *Voyager 2* observations and the current states of their dynamos are not well understood. Single-pass flyby ocean detection and characterization is possible, but requires the use of a planetary magnetic field model, as it is otherwise nearly impossible to adequately characterize the long-period excitations on a single flyby [23]. For a two-flyby trajectory, the vector observations acquired during the two passes of the moon are insufficient to uniquely characterize both the static background field and the synodic excitation of the planetary field as three model parameters are required for each component. However, a three-flyby mission concept provides the additional planetary field observation needed to uniquely characterize these three parameters for each vector component. If multiple excitations are present with different periods (e.g. the second harmonic of the synodic) the trajectory should be designed such that each flyby uniformly samples the phase for each period. In this case, more than one planetary field excitation can be characterized, even with the use of a three-flyby trajectory.

Fortunately, for the Uranus system, as shown, three flybys of the moon suffice to characterize the static background field and the excitations associated with the synodic and its second harmonic. To further demonstrate this point, we include the results for the two-flyby case of Umbriel in the Appendix. Insufficient characterization of the driving field leads to erroneous biases in the estimates of the induction coefficients, which compromises the assessment of the interior structure, including the ocean.

## 5. Conclusion

In summary, we have demonstrated a distance-based ensemble modelling approach that allows for subsurface ocean detection and characterization using magnetic induction observations. The Monte Carlo uncertainty quantification method we employ provides a robust means to determine the uncertainty of the moon interior parameters and, therefore, the constraints that can be placed on ice-shell thickness, ocean thickness and electrical conductivity. These methods are shown to be of use for characterizing a theoretical ocean within the moon of Umbriel. They have also been applied to assessing *Galileo* magnetometer measurements at Callisto, and evaluated for application to other moons in the solar system, including Triton. The methods would also be useful for assessing observations acquired by the *Europa Clipper* and *JUICE* missions to the Jovian moon system and should also be useful for trajectory design and formulation of NASA’s highly prioritized *Uranus Orbiter and Probe* mission.

## 6. Open research

For all timing and ephemeris computation in this work, the NAIF developed SPICE toolkit [58] for MATLAB called ‘mice’ was utilized, freely available at https://naif.jpl.nasa.gov/naif/. All magnetic field models and field line visuals were implemented in MATLAB. The most recent kernel files were leveraged and all IAU defined reference frames defined in the IAU working group report [59] were used. The forward models used in this work are the same used in [21] and archived on Zenodo [60]. The Urania trajectory of the three flybys of Umbriel are included in the supplemental material in ascii format. In increasing order of column number are the Coordinated Universal Time (UTC), seconds past J2000, spacecraft position (*x*, *y* and *z*) with respect to the centre of Uranus in the IAU Uranus frame in units of Uranus radii (25559km), and spacecraft position (*x*, *y* and *z*) with respect to the centre of Umbriel in the IAU Umbriel frame in units of Umbriel radii (584.7km).

## Figures and Tables

**Figure 9.**
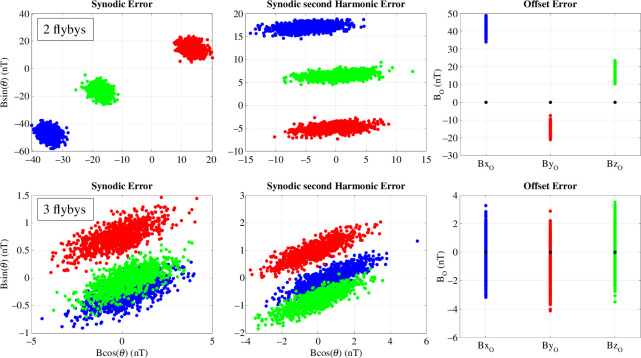
Results of the planetary field inversion of (top row) two-flyby and (bottom row) three-flyby cases. From left-to-right, the panels represent the error in the synodic period ($B_{x}$ in blue, $B_{y}$ in red and $B_{z}$ in green), the error in the synodic second harmonic, the error in the offset and the reconstructed planetary field for each case. Although the reconstruction appears to have a good fit, two flybys are not sufficient to characterize the excitations, as apparent from the biases that are introduced. Conversely, for the three-flyby case, the mean of the error distributions are very close to zero. This is simply because the synodic period is sampled with sufficient spread in phase space over the three flybys to accurately fit the sinusoidal components of the excitations.

**Figure 10.**
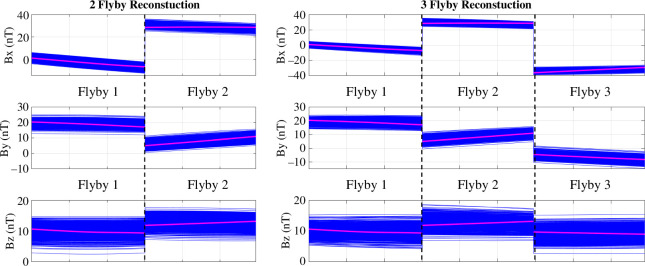
Reconstruction of the planetary field using (left) two and (right) three flybys. The magenta lines represent the true planetary field, simulated with a magnetic field model, where the different segments representing the $t_{ca}\pm$ 20 min data segments used for each flyby to model the field. The swath of blue lines represents the reconstructed planetary field model (i.e. equation (2.5)) from all 1000 Monte Carlo simulations.

**Figure 11.**
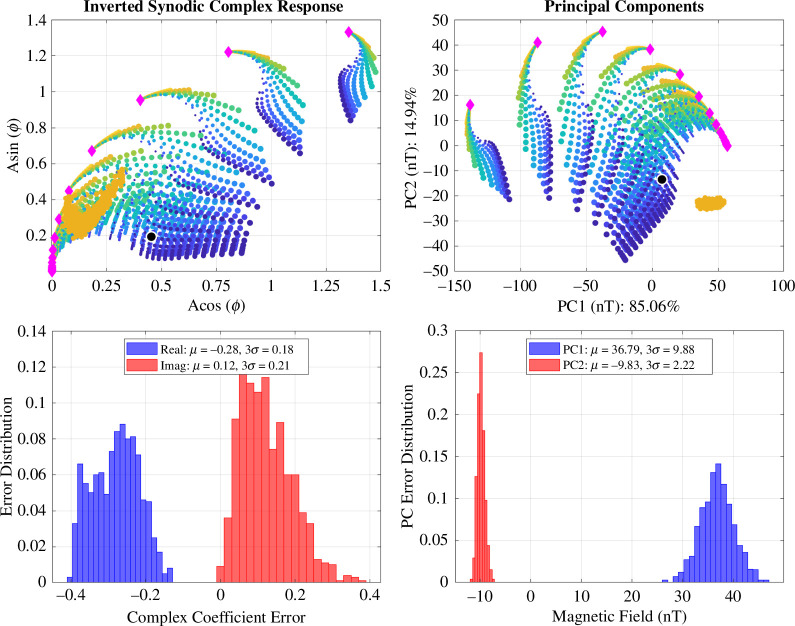
Results of the inversion and PCA Monte Carlo analysis for Umbriel for the two-flyby case. (upper left) Inverted synodic complex responses from the Monte Carlo simulation (orange), plotted on top of synodic complex response from all forward models used in this study. The black dot represents the true model response used in this study for reference. (bottom left) Errors associated with each iteration of the Monte Carlo simulation from the inversion approach. (upper right) PCs associated with the Monte Carlo simulation (orange), plotted on top of the PCs from all forward models used in this study. The black dot again represents the true model response used in this study for reference. (bottom left) Errors associated with each iteration of the Monte Carlo simulation from the PCA approach.

## Data Availability

This work contains a dataset that was used for a previously published paper [21]. The data are already publicly accessible in Zendo [60]. Supplementary material is available online [61].

## Appendix A

### (a) Linear equations of magnetic induction coefficients

In the following, we illustrate the linear relationship of the induction coefficients and describe our implementation for a conventional linear least-squares solution.

The induced field contribution at the spacecraft, modelled as ${\hat{𝐁}}_{\mathrm{Ind}}(t)=𝐃(t)\hat{𝐦}(t)$, where the model parameter $\hat{𝐦}(t)$ (i.e. time-varying portion of the induced magnetic moment) changes on every flyby, can be expanded to

(A 1)
$${\hat{B}}_{Ind}(t)\approx D(t)P(t)A.$$

Here, 𝐃(t) is the time-dependent dipolar matrix which mixes the magnetic moment components into each axis of the magnetic field and 𝐏(t) is the time-dependent matrix that contains the planetary driving field coefficients ${𝐁}_{R,k}$ and ${𝐁}_{I,k}$ for each flyby, both defined later in the appendix. We define 𝐀 as an array that contains the real and imaginary complex coefficients for the frequencies used, $\left[A_{R,1},A_{I,1},\ldots ,A_{R,K},A_{I,K}\right]$, which are the same for every flyby and linearly separable from the time-dependent terms. Therefore, induction observations acquired from every flyby can be used jointly to estimate the complex response function of the induced response of the ocean at multiple frequencies by minimizing the following error function:

(A 2)
$$\underset{A_{R,k},A_{I,k}}{\arg\min}\sum_{t}{\left({\tilde{B}}_{Ind}(t)-D(t)P(t)A\right)}^{2},$$

where ${\tilde{B}}_{Ind}(t)$ is the approximation of the induced field extracted from the observations (i.e. observations with background planetary field subtracted). We use a constrained linear optimization to ensure both real and imaginary complex coefficients stay in the range 0–1.5, consistent with the complex space illustrated in figure 2.

To begin, we expand the time-varying portion of the magnetic moment of equation (2.6):

(A 3)
$$\begin{array}{rl} {\hat{m}}_{x}(t) & =\sum_{k}A_{k}B_{x,k}\cos\left(2\pi f_{k}t+\theta_{x,k}-\phi_{k}\right), \end{array}$$

(A 4)
$$\begin{array}{rl} {\hat{m}}_{y}(t) & =\sum_{k}A_{k}B_{y,k}\cos\left(2\pi f_{k}t+\theta_{y,k}-\phi_{k}\right), \end{array}$$

(A 5)
$$\begin{array}{cccc} & {\hat{m}}_{z}(t) & =\sum_{k}A_{k}B_{z,k}\cos\left(2\pi f_{k}t+\theta_{z,k}-\phi_{k}\right). & \end{array}$$

Using the following identities:

(A 6)
$$\begin{array}{rl} A\cos(\omega t-\theta ) & =A\cos\theta \cos\omega t+A\sin\theta \sin\omega t, \end{array}$$

(A 7)
$$\begin{array}{cccc} & A\;\sin(\omega t-\theta ) & =-A\;\sin\;\theta \;\cos\;\omega t+A\;\cos\;\theta \;\sin\;\omega t, & \end{array}$$

the equations can be expanded into

(A 8)
$${\hat{m}}_{x}(t)=\sum_{k}A_{R,k}B_{R,x,k}\cos(\omega_{k}t)+A_{R,k}B_{I,x,k}\sin(\omega_{k}t)-A_{I,k}B_{I,x,k}\cos(\omega_{k}t)+A_{I,k}B_{R,x,k}\sin(\omega_{k}t),$$

(A 9)
$${\hat{m}}_{y}(t)=\sum_{k}A_{R,k}B_{R,y,k}\cos(\omega_{k}t)+A_{R,k}B_{I,y,k}\sin(\omega_{k}t)-A_{I,k}B_{I,y,k}\cos(\omega_{k}t)+A_{I,k}B_{R,y,k}\sin(\omega_{k}t),$$

(A 10)
$$\begin{array}{cccc} & {\hat{m}}_{z}(t) & =\sum_{k}A_{R,k}B_{R,z,k}\cos(\omega_{k}t)+A_{R,k}B_{I,z,k}\sin(\omega_{k}t)-A_{I,k}B_{I,z,k}\cos(\omega_{k}t)+A_{I,k}B_{R,z,k}\sin(\omega_{k}t), & \end{array}$$

where the planetary driving field coefficients are defined by

(A 11)
$$B_{R,x,k}=B_{x,k}\cos(\theta_{x,k}),\;B_{I,x,k}=B_{x,k}\sin(\theta_{x,k}),$$

(A 12)
$$B_{R,y,k}=B_{y,k}\cos(\theta_{y,k}),\;B_{I,y,k}=B_{y,k}\sin(\theta_{y,k}),$$

(A 13)
$$B_{R,z,k}=B_{z,k}\cos(\theta_{z,k}),\;B_{I,z,k}=B_{z,k}\sin(\theta_{z,k}),$$

and the induction coefficients are defined by

(A 14)
$$\begin{array}{ccc} & A_{R,k}=A_{k}\cos(\phi_{k})\;A_{I,k}=A_{k}\sin(\phi_{k}). & \end{array}$$

In matrix form, the magnetic moment at time t can be written as

(A 15)
$$\begin{array}{ccc} & \hat{𝐦}(t)=\left[{𝐁}_{U,R}⊙{𝐒}_{1}(t)+{𝐁}_{U,I}⊙{𝐒}_{2}(t)\right]𝐀=𝐏(t)𝐀, & \end{array}$$

where

(A 16)
$$\begin{array}{rl} B_{U,R} & =\left[\begin{array}{cccc} B_{R,x,1} & B_{R,x,1} & B_{R,x,2} & B_{R,x,2}\ldots \\ B_{R,y,1} & B_{R,y,1} & B_{R,y,2} & B_{R,y,2}\ldots \\ B_{R,z,1} & B_{R,z,1} & B_{R,z,2} & B_{R,z,2}\ldots \end{array}\right], \end{array}$$

(A 17)
$$\begin{array}{rl} B_{U,I} & =\left[\begin{array}{cccc} B_{I,x,1} & B_{I,x,1} & B_{I,x,2} & B_{I,x,2}\ldots \\ B_{I,y,1} & B_{I,y,1} & B_{I,y,2} & B_{I,y,2}\ldots \\ B_{I,z,1} & B_{I,z,1} & B_{I,z,2} & B_{I,z,2}\ldots \end{array}\right], \end{array}$$

(A 18)
$$\begin{array}{rl} S_{1}(t) & =\left[\begin{array}{cccc} \cos(\omega_{1}t) & \sin(\omega_{1}t) & \cos(\omega_{2}t) & \sin(\omega_{2}t)\ldots \\ \cos(\omega_{1}t) & \sin(\omega_{1}t) & \cos(\omega_{2}t) & \sin(\omega_{2}t)\ldots \\ \cos(\omega_{1}t) & \sin(\omega_{1}t) & \cos(\omega_{2}t) & \sin(\omega_{2}t)\ldots \end{array}\right], \end{array}$$

(A 19)
$$\begin{array}{rl} S_{2}(t) & =\left[\begin{array}{cccc} \sin(\omega_{1}t) & -\cos(\omega_{1}t) & \sin(\omega_{2}t) & -\cos(\omega_{2}t)\ldots \\ \sin(\omega_{1}t) & -\cos(\omega_{1}t) & \sin(\omega_{2}t) & -\cos(\omega_{2}t)\ldots \\ \sin(\omega_{1}t) & -\cos(\omega_{1}t) & \sin(\omega_{2}t) & -\cos(\omega_{2}t)\ldots \end{array}\right] \end{array}$$

are all matrices of 3×2K in size and ⊙ represents matrix-element-wise product (also known as a Hadamard product). Here, the induction coefficients, which dictate ocean properties, are linearly separable.

The induced magnetic field at time t (${𝐁}_{\mathrm{Ind}}(t)=[B_{\mathrm{Ind},x}(t)$, $B_{\mathrm{Ind},y}(t)$, $B_{\mathrm{Ind},z}(t)]$) can be expressed as a matrix multiplication of the dipolar matrix 𝐃(t) and the time-varying portion of the magnetic moment matrix 𝐦(t):

(A 20)
$$\begin{array}{cccc} & {𝐁}_{\mathrm{Ind}}(t) & =𝐃(t)𝐦(t), & \end{array}$$

(A 21)
$$\left[\begin{array}{c} B_{Ind,x}(t)\\ B_{Ind,y}(t)\\ B_{Ind,z}(t) \end{array}\right]=\left[\begin{array}{ccc} D_{11}(t) & D_{12}(t) & D_{13}(t)\\ D_{21}(t) & D_{22}(t) & D_{23}(t)\\ D_{31}(t) & D_{32}(t) & D_{33}(t) \end{array}\right]\left[\begin{array}{c} m_{x}(t)\\ m_{y}(t)\\ m_{z}(t) \end{array}\right],$$

where the elements of the 3-by-3 dipolar matrix are defined by

(A 22)
$$\begin{array}{ccc} & \begin{array}{c} 𝐃(t)=-\frac{R^{3}}{2}\frac{1}{r(t{)}^{5}}\left[\begin{array}{ccc} 3x(t{)}^{2}-r(t{)}^{2} & 3x(t)y(t) & 3x(t)z(t)\\ 3y(t)x(t) & 3y(t{)}^{2}-r(t{)}^{2} & 3y(t)z(t)\\ 3z(t)x(t) & 3z(t)y(t) & 3z(t{)}^{2}-r(t{)}^{2} \end{array}\right] \end{array} & \end{array}$$

and x(t),y(t),z(t),r(t) define the position of the spacecraft at time t in the frame of the moon.

If it is assumed that the magnetic moment is static for each flyby owing to their slow variation compared with the spacecraft velocity, then $𝐦(t)\approx \hat{𝐦}(t_{ca})=𝐏(t_{ca})𝐀$, where $t_{ca}$ is the time at closest approach for the flyby. In this case, we can define the following matrices for each flyby:

(A 23)
$$D_{x}=\left[\begin{array}{ccc} D_{11}(t_{1}) & D_{12}(t_{1}) & D_{13}(t_{1})\\ \vdots & \vdots & \vdots \\ D_{11}(t_{N}) & D_{12}(t_{N}) & D_{13}(t_{N}) \end{array}\right]P(t_{ca}),$$

(A 24)
$$D_{y}=\left[\begin{array}{ccc} D_{21}(t_{1}) & D_{22}(t_{1}) & D_{23}(t_{1})\\ \vdots & \vdots & \vdots \\ D_{21}(t_{N}) & D_{22}(t_{N}) & D_{23}(t_{N}) \end{array}\right]P(t_{ca}),$$

(A 25)
$$\begin{array}{cccc} & {𝐃}_{z} & =\left[\begin{array}{ccc} D_{31}(t_{1}) & D_{32}(t_{1}) & D_{33}(t_{1})\\ \vdots \; & \vdots \; & \vdots \;\\ D_{31}(t_{N}) & D_{32}(t_{N}) & D_{33}(t_{N}) \end{array}\right]𝐏(t_{ca}) & \end{array}$$

and

(A 26)
$$\begin{array}{ccc} & 𝐃=\left[\begin{array}{c} {𝐃}_{x}\\ {𝐃}_{y}\\ {𝐃}_{z} \end{array}\right], & \end{array}$$

which is a matrix of size 3N×2K, where K represents the number of frequencies in the model and N represents the number of vectors that were measured during the flyby, here within $5R_{M}$ (where $R_{M}$ is the moon radius), sufficient to capture the $1/r^{3}$ fall-off of the induced dipole field.

If observations from F flybys are used, the matrix can be expanded as

(A 27)
$$\begin{array}{ccc} & {𝐃}_{F}=\left[\begin{array}{c} {𝐃}_{x,1}\\ \vdots \;\\ {𝐃}_{x,F}\\ {𝐃}_{y,1}\\ \vdots \;\\ {𝐃}_{y,F}\\ {𝐃}_{z,1}\\ \vdots \;\\ {𝐃}_{z,F} \end{array}\right] & \end{array}$$

and the observations are stored as

(A 28)
$${\tilde{B}}_{Ind,F}=[{\tilde{B}}_{Ind,x}(t_{1}),\ldots {\tilde{B}}_{Ind,x}(t_{NF}),{\tilde{B}}_{Ind,y}(t_{1}),\ldots {\tilde{B}}_{Ind,y}(t_{NF}),{\tilde{B}}_{Ind,z}(t_{1}),\ldots {\tilde{B}}_{Ind,z}(t_{NF}){]}^{T},$$

which is identical to the concatenation of the observation array used for PCA in equation (2.8). Stacking the observations and matrices from each of the F flybys in this fashion allows for one to solve for a single set of complex response function coefficients 𝐀 that is optimum across all the flybys. Here, the magnetic induction field estimates can written in compact notation,

(A 29)
$$\begin{array}{ccc} & {\hat{𝐁}}_{\mathrm{Ind},F}={𝐃}_{F}𝐀. & \end{array}$$

The induction coefficients 𝐀 associated with the K frequencies can then be solved directly with least-squares estimate

(A 30)
$$A={\left[D_{F}^{T}D_{F}\right]}^{-1}D_{F}^{T}{\tilde{B}}_{Ind,F},$$

which can be implemented to have constraints within the complex response plane, e.g. $0<A_{R,k},A_{I,k}<1.5$. Note that this equation is the solution to minimizing the error of the function in equation (A 2). Note also the similarity between this equation and the projection of the data of the PCA approach in equations (2.9) and (A 21), where here, the psuedo-inverse matrix $[{𝐃}_{F}^{T}{𝐃}_{F}{]}^{-1}{𝐃}_{F}^{T}$ acts very similar to the eigenvalues of the PCA method. For simplicity of description, if we assume each flyby contains N vectors, then the array of estimates ${\hat{𝐁}}_{\mathrm{Ind},F}$ is 3NF×1 in size, ${𝐃}_{F}$ is 3NF×2K in size, and 𝐀 is 2K×1 in size. The reconstructed induced dipole magnetic field is simply computed at time t by

(A 31)
$$\begin{array}{ccc} & {\hat{𝐁}}_{\mathrm{Ind}}(t)=𝐃(t)𝐏(t)𝐀, & \end{array}$$

and can be added to the modelled planetary field for the full reconstruction.

## Appendix B

### (b) The need for three flybys

Here, we show the necessity for the trajectory have at least three flybys if there is any desire to gain insight into the properties of the ocean, aside from simply detecting its presence. Because there is ambiguity in the complexity of Uranus’ magnetic field owing to the limited measurements that were acquired from *Voyager 2* during its single pass of the planet, specifically the quadrupole and octopole moments which appear as the higher-order harmonics in the frame of the moon, it cannot be assumed that the wave associated with the synodic second harmonic period is negligible. However, the time variation of the magnetic field at the period of Umbriel’s orbit is expected to be weak owing to its near-circular orbit so is not included in the inversion. Therefore, the planetary field model involves six parameters associated with the synodic excitation (amplitudes and phase for each magnetic component), six parameters associated with the second harmonic of the synodic excitation and three offsets, totalling 15 parameters. Figure 9 illustrates the errors associated with the recovery of these planetary field coefficients for two and three flyby cases. As illustrated, because there is an insufficient number of observations (i.e. the system is underdetermined), the error distributions acquired from the 1000 Monte Carlo simulations exhibit large systematic errors that manifest as offsets in the parameters, even though the estimated model appears to sufficiently reconstruct the planetary field for these two flybys as illustrated in figure 10.

Figure 11 illustrates the effect that these biases in planetary field errors have on the assessment of the interior properties after the two flybys of Umbriel. As illustrated, offsets in the driving field planetary field manifest as offsets in the classification space, leading to erroneous interior parameter assessment.
